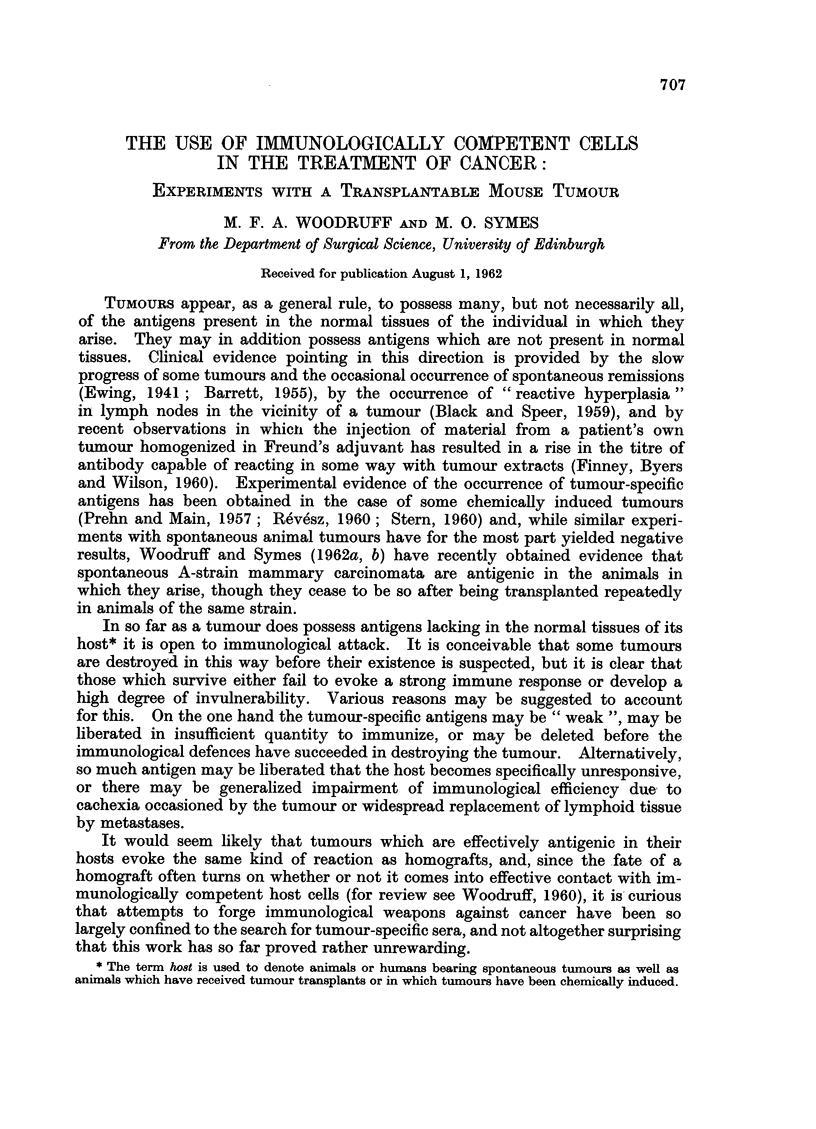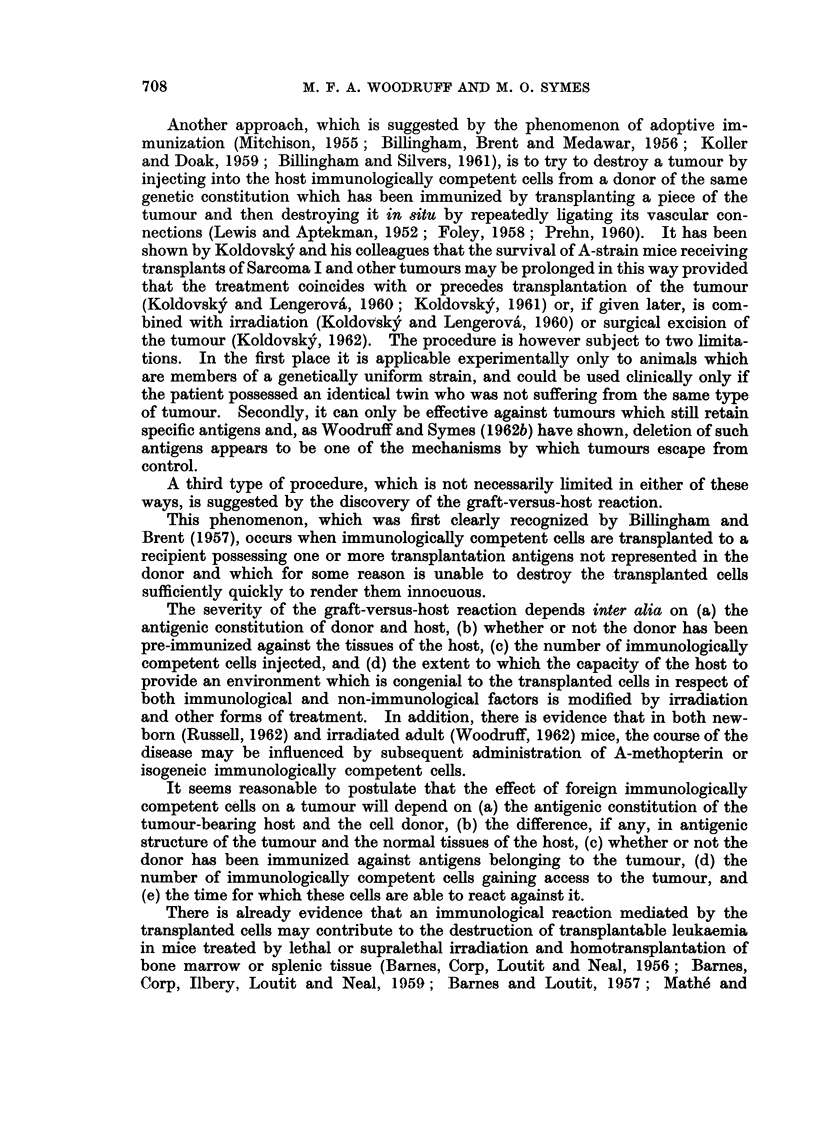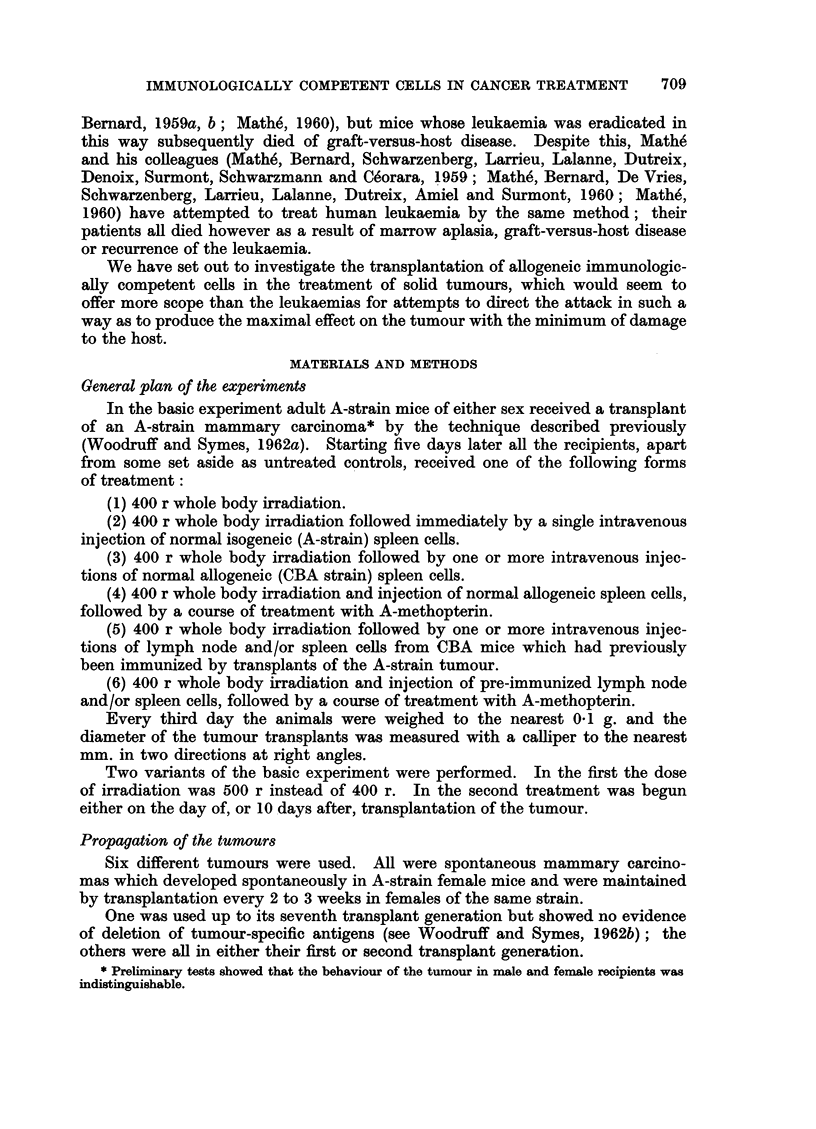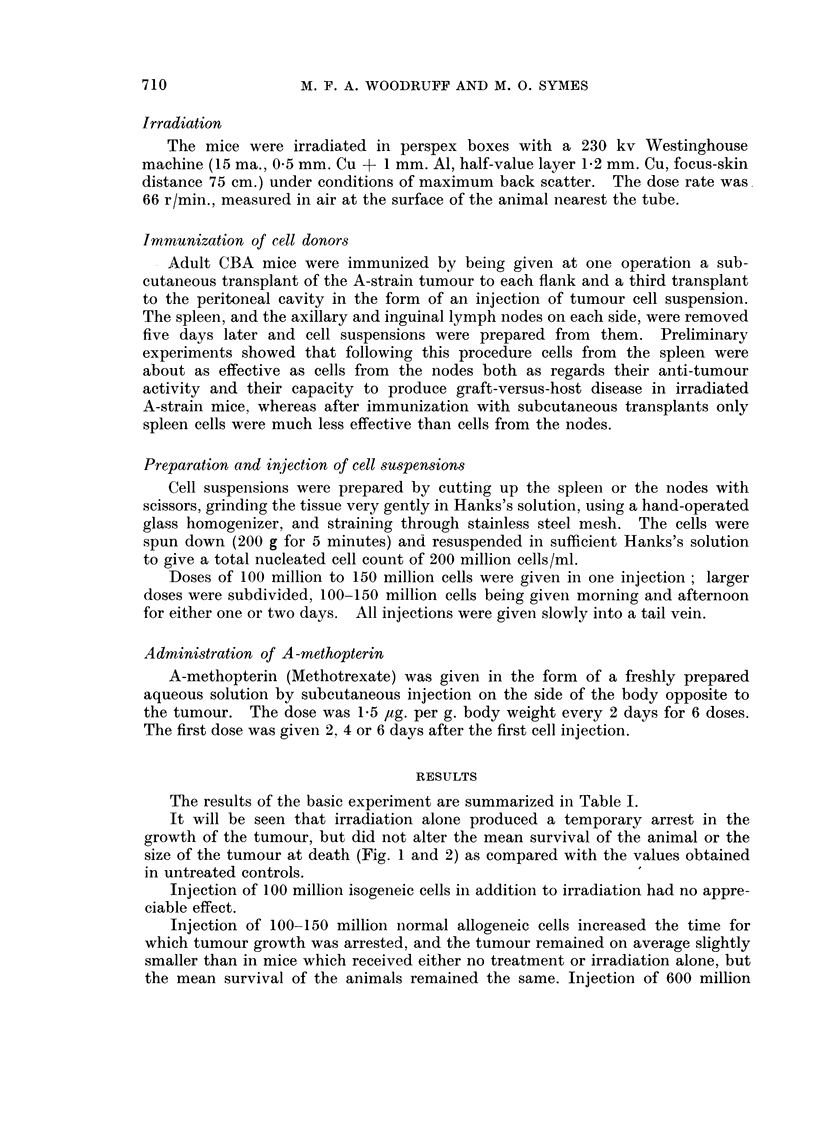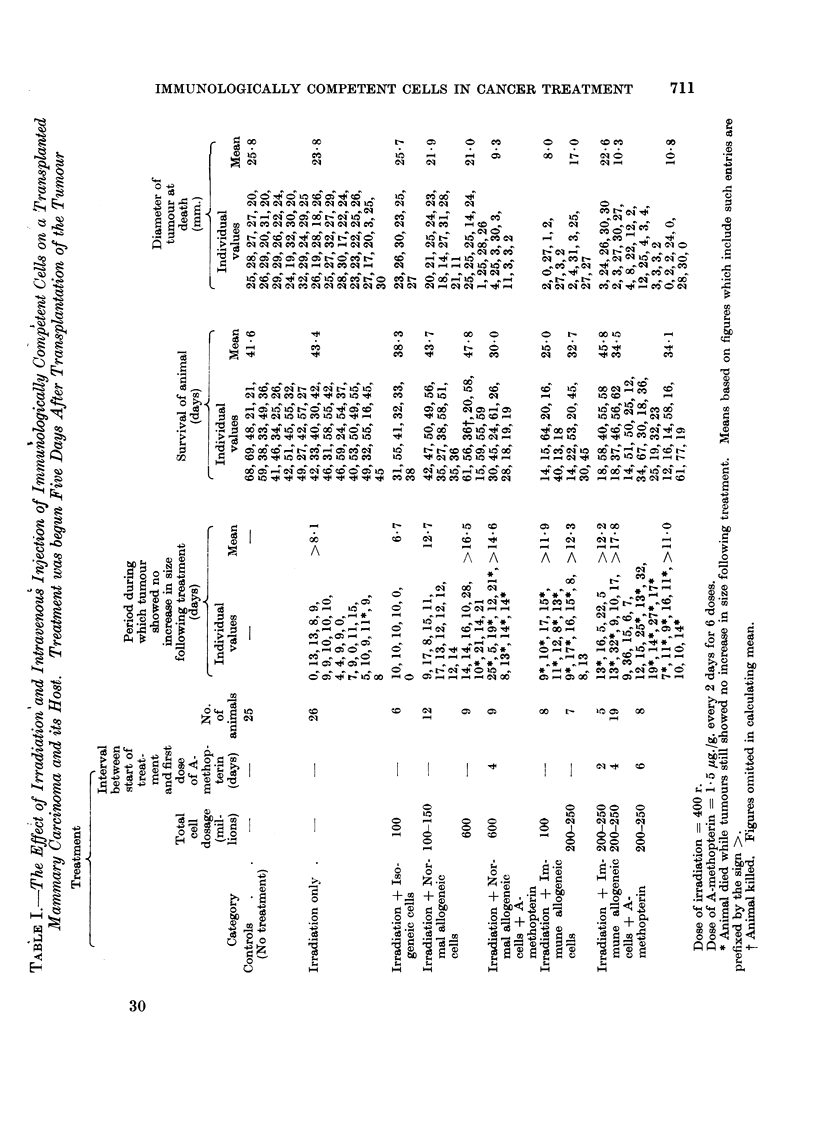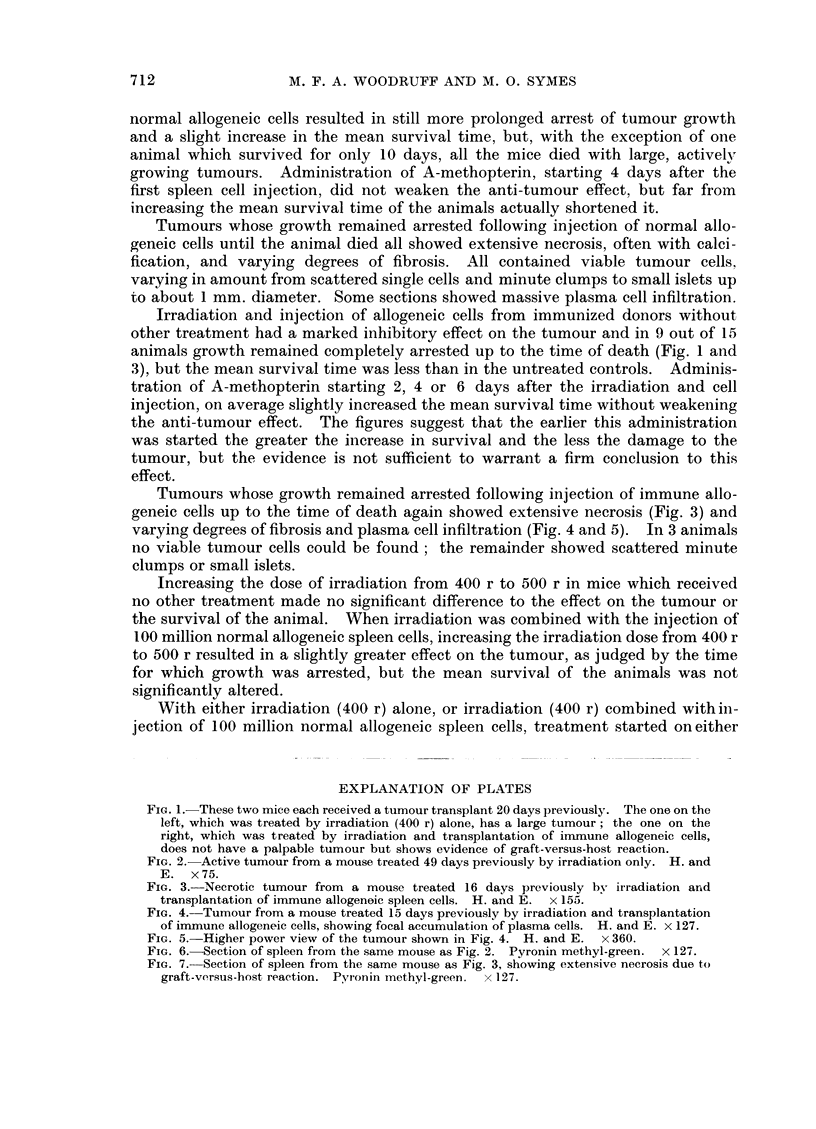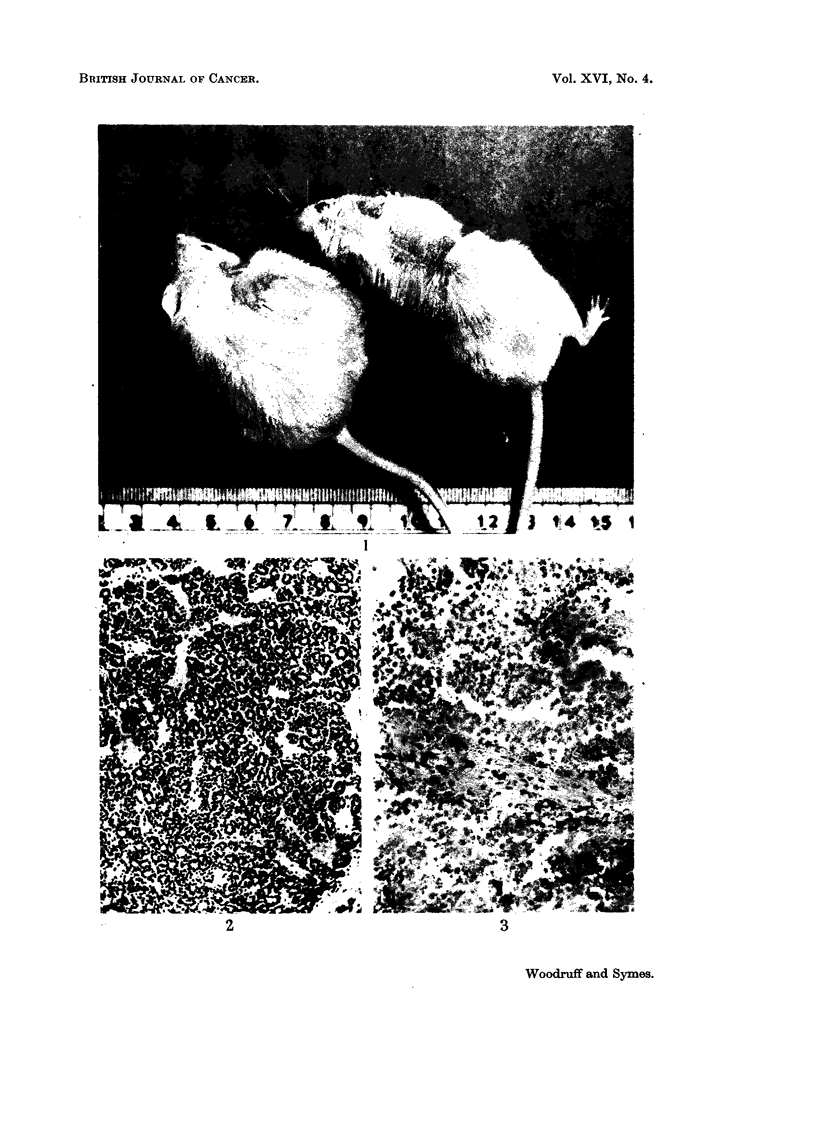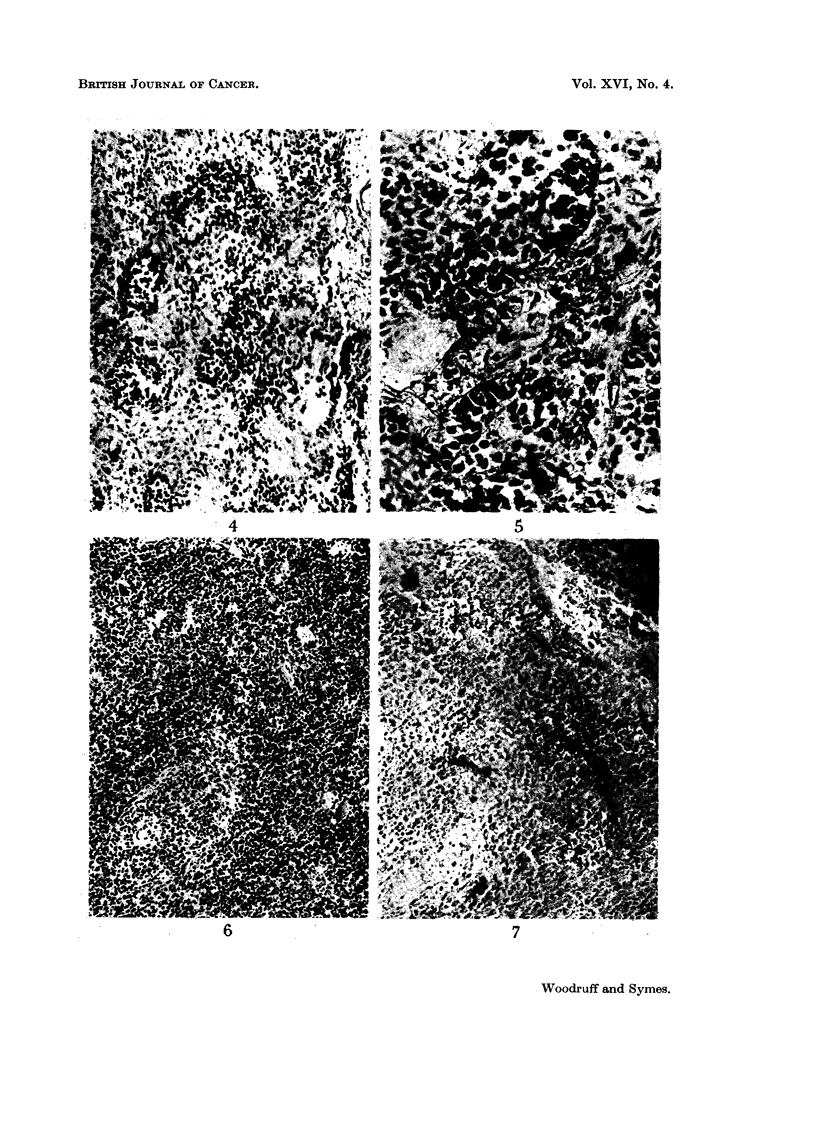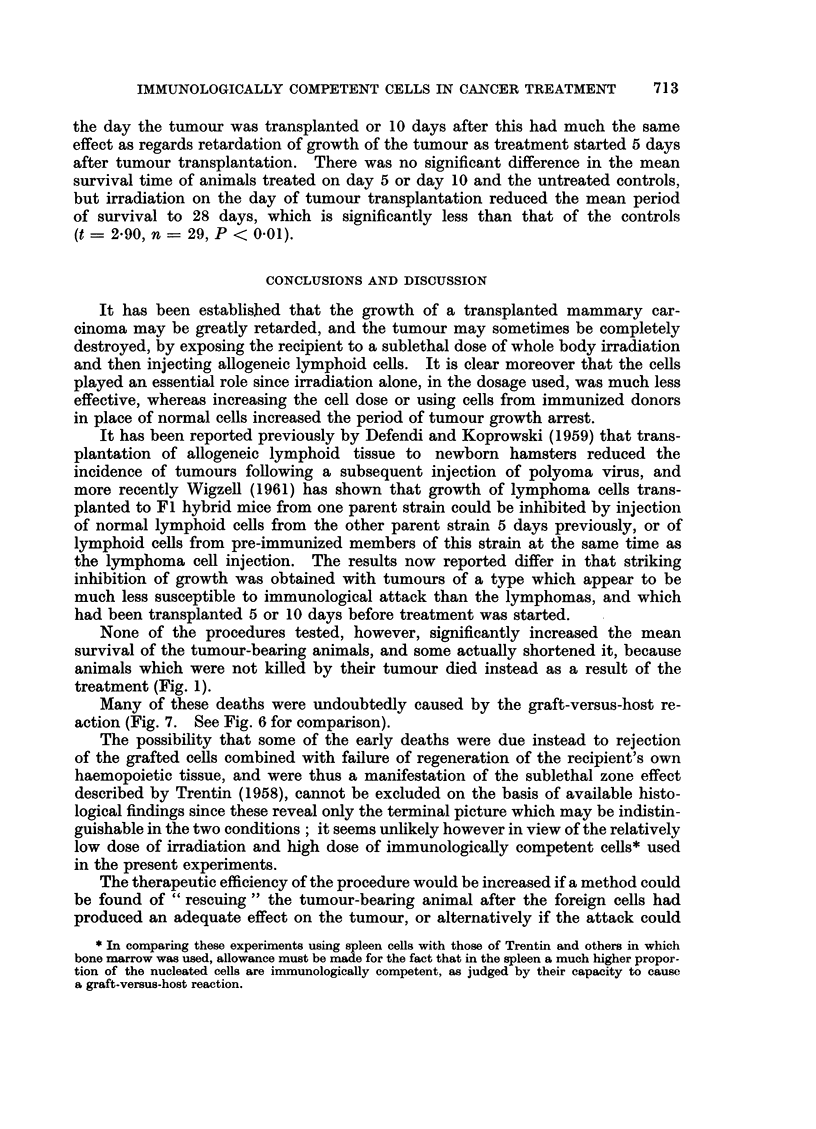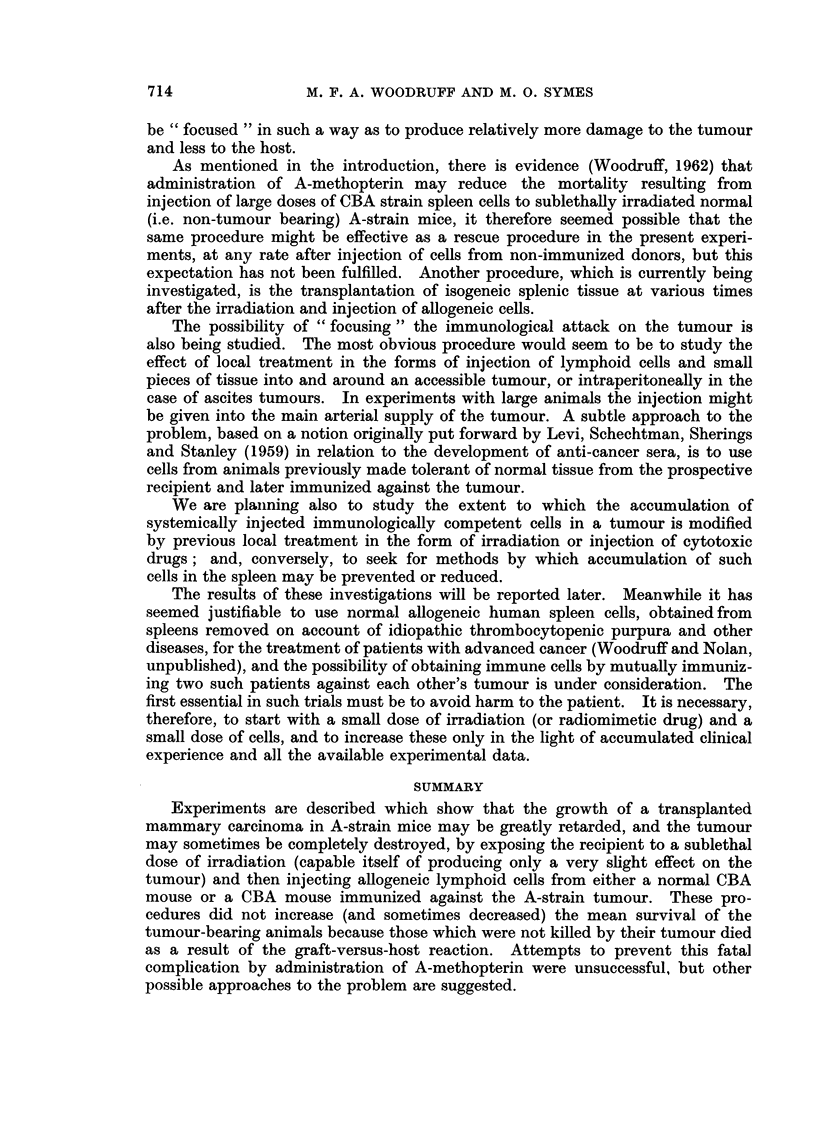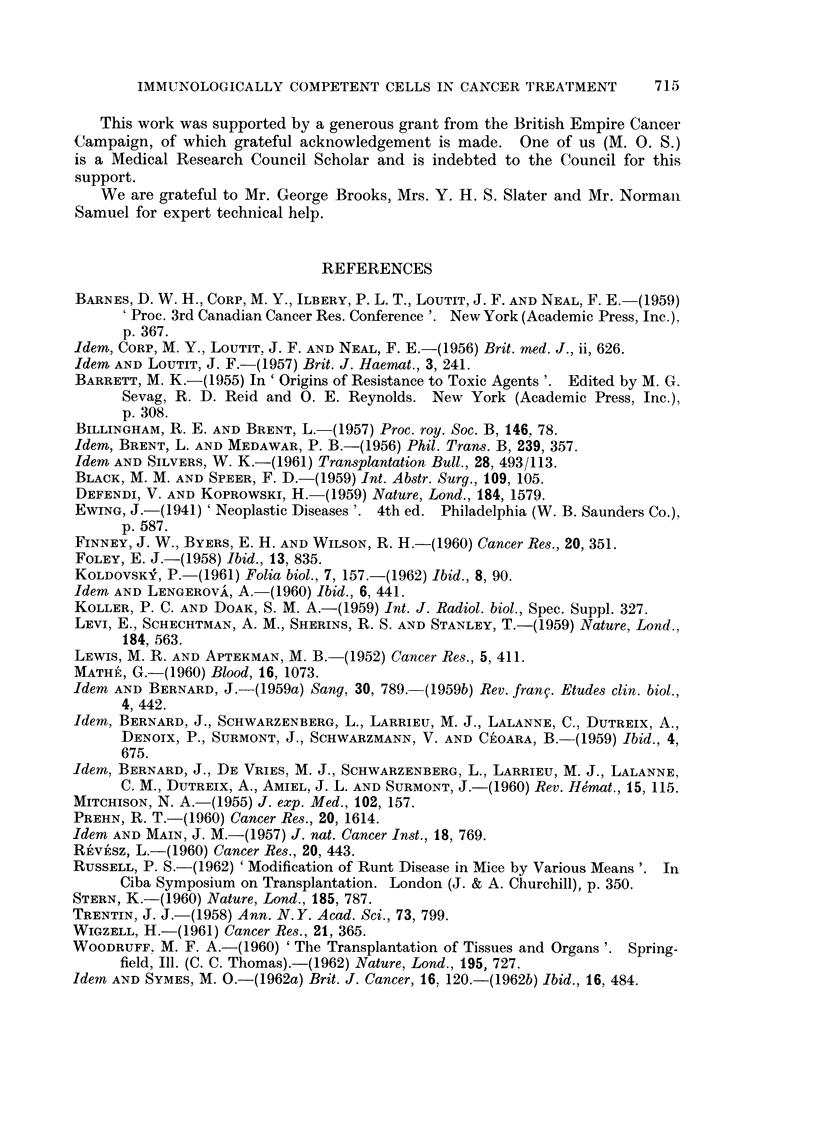# The Use of Immunologically Competent Cells in the Treatment of Cancer

**DOI:** 10.1038/bjc.1962.82

**Published:** 1962-12

**Authors:** M. F. A. Woodruff, M. O. Symes

## Abstract

**Images:**


### THE USE OF IMMUNOLOGICALLY COMPETENT CELLS IN THE TREATMENT OF CANCER:

### EXPERIMENTS WITH A TRANSPLANTABLE MOUSE TUMOUR

M. F. A. WOODRUFF AND M. O. SYMES

*From the Department of Surgical Science, University of Edinburgh*

Received for publication August 1, 1962

TUMOURS appear, as a general rule, to possess many, but not necessarily all, of the antigens present in the normal tissues of the individual in which they arise. They may in addition possess antigens which are not present in normal tissues. Clinical evidence pointing in this direction is provided by the slow progress of some tumours and the occasional occurrence of spontaneous remissions (Ewing, 1941 ; Barrett, 1955), by the occurrence of " reactive hyperplasia " in lymph nodes in the vicinity of a tumour (Black and Speer, 1959), and by recent observations in which the injection of material from a patient's own tumour homogenized in Freund's adjuvant has resulted in a rise in the titre of antibody capable of reacting in some way with tumour extracts (Finney, Byers and Wilson, 1960). Experimental evidence of the occurrence of tumour-specific antigens has been obtained in the case of some chemically induced tumours (Prehn and Main, 1957 ; Révész, 1960 ; Stern, 1960) and, while similar experiments with spontaneous animal tumours have for the most part yielded negative results, Woodruff and Symes (1962*a*, *b*) have recently obtained evidence that spontaneous A-strain mammary carcinomata are antigenic in the animals in which they arise, though they cease to be so after being transplanted repeatedly in animals of the same strain.

In so far as a tumour does possess antigens lacking in the normal tissues of its host* it is open to immunological attack. It is conceivable that some tumours are destroyed in this way before their existence is suspected, but it is clear that those which survive either fail to evoke a strong immune response or develop a high degree of invulnerability. Various reasons may be suggested to account for this. On the one hand the tumour-specific antigens may be " weak ", may be liberated in insufficient quantity to immunize, or may be deleted before the immunological defences have succeeded in destroying the tumour. Alternatively, so much antigen may be liberated that the host becomes specifically unresponsive, or there may be generalized impairment of immunological efficiency due to cachexia occasioned by the tumour or widespread replacement of lymphoid tissue by metastases.

It would seem likely that tumours which are effectively antigenic in their hosts evoke the same kind of reaction as homografts, and, since the fate of a homograft often turns on whether or not it comes into effective contact with immunologically competent host cells (for review see Woodruff, 1960), it is curious that attempts to forge immunological weapons against cancer have been so largely confined to the search for tumour-specific sera, and not altogether surprising that this work has so far proved rather unrewarding.

* The term *host* is used to denote animals or humans bearing spontaneous tumours as well as animals which have received tumour transplants or in which tumours have been chemically induced.

Another approach, which is suggested by the phenomenon of adoptive immunization (Mitchison, 1955; Billingham, Brent and Medawar, 1956; Koller and Doak, 1959; Billingham and Silvers, 1961), is to try to destroy a tumour by injecting into the host immunologically competent cells from a donor of the same genetic constitution which has been immunized by transplanting a piece of the tumour and then destroying it *in situ* by repeatedly ligating its vascular connections (Lewis and Aptekman, 1952; Foley, 1958; Prehn, 1960). It has been shown by Koldovský and his colleagues that the survival of A-strain mice receiving transplants of Sarcoma I and other tumours may be prolonged in this way provided that the treatment coincides with or precedes transplantation of the tumour (Koldovský and Lengerová, 1960; Koldovský, 1961) or, if given later, is combined with irradiation (Koldovský and Lengerová, 1960) or surgical excision of the tumour (Koldovský, 1962). The procedure is however subject to two limitations. In the first place it is applicable experimentally only to animals which are members of a genetically uniform strain, and could be used clinically only if the patient possessed an identical twin who was not suffering from the same type of tumour. Secondly, it can only be effective against tumours which still retain specific antigens and, as Woodruff and Symes (1962*b*) have shown, deletion of such antigens appears to be one of the mechanisms by which tumours escape from control.

A third type of procedure, which is not necessarily limited in either of these ways, is suggested by the discovery of the graft-versus-host reaction.

This phenomenon, which was first clearly recognized by Billingham and Brent (1957), occurs when immunologically competent cells are transplanted to a recipient possessing one or more transplantation antigens not represented in the donor and which for some reason is unable to destroy the transplanted cells sufficiently quickly to render them innocuous.

The severity of the graft-versus-host reaction depends *inter alia* on (a) the antigenic constitution of donor and host, (b) whether or not the donor has been pre-immunized against the tissues of the host, (c) the number of immunologically competent cells injected, and (d) the extent to which the capacity of the host to provide an environment which is congenial to the transplanted cells in respect of both immunological and non-immunological factors is modified by irradiation and other forms of treatment. In addition, there is evidence that in both newborn (Russell, 1962) and irradiated adult (Woodruff, 1962) mice, the course of the disease may be influenced by subsequent administration of A-methopterin or isogeneic immunologically competent cells.

It seems reasonable to postulate that the effect of foreign immunologically competent cells on a tumour will depend on (a) the antigenic constitution of the tumour-bearing host and the cell donor, (b) the difference, if any, in antigenic structure of the tumour and the normal tissues of the host, (c) whether or not the donor has been immunized against antigens belonging to the tumour, (d) the number of immunologically competent cells gaining access to the tumour, and (e) the time for which these cells are able to react against it.

There is already evidence that an immunological reaction mediated by the transplanted cells may contribute to the destruction of transplantable leukaemia in mice treated by lethal or supralethal irradiation and homotransplantation of bone marrow or splenic tissue (Barnes, Corp, Loutit and Neal, 1956; Barnes, Corp, Ilbery, Loutit and Neal, 1959; Barnes and Loutit, 1957; Mathé and

Bernard, 1959*a*, *b* ; Mathé, 1960), but mice whose leukaemia was eradicated in this way subsequently died of graft-versus-host disease. Despite this, Mathé and his colleagues (Mathé, Bernard, Schwarzenberg, Larrieu, Lalanne, Dutreix, Denoix, Surmont, Schwarzmann and Céorara, 1959 ; Mathé, Bernard, De Vries, Schwarzenberg, Larrieu, Lalanne, Dutreix, Amiel and Surmont, 1960 ; Mathé, 1960) have attempted to treat human leukaemia by the same method ; their patients all died however as a result of marrow aplasia, graft-versus-host disease or recurrence of the leukaemia.

We have set out to investigate the transplantation of allogeneic immunologically competent cells in the treatment of solid tumours, which would seem to offer more scope than the leukaemias for attempts to direct the attack in such a way as to produce the maximal effect on the tumour with the minimum of damage to the host.

### MATERIALS AND METHODS

### *General plan of the experiments*

In the basic experiment adult A-strain mice of either sex received a transplant of an A-strain mammary carcinoma* by the technique described previously (Woodruff and Symes, 1962*a*). Starting five days later all the recipients, apart from some set aside as untreated controls, received one of the following forms of treatment :

(1) 400 r whole body irradiation.

(2) 400 r whole body irradiation followed immediately by a single intravenous injection of normal isogeneic (A-strain) spleen cells.

(3) 400 r whole body irradiation followed by one or more intravenous injections of normal allogeneic (CBA strain) spleen cells.

(4) 400 r whole body irradiation and injection of normal allogeneic spleen cells, followed by a course of treatment with A-methopterin.

(5) 400 r whole body irradiation followed by one or more intravenous injections of lymph node and/or spleen cells from CBA mice which had previously been immunized by transplants of the A-strain tumour.

(6) 400 r whole body irradiation and injection of pre-immunized lymph node and/or spleen cells, followed by a course of treatment with A-methopterin.

Every third day the animals were weighed to the nearest 0·1 g. and the diameter of the tumour transplants was measured with a calliper to the nearest mm. in two directions at right angles.

Two variants of the basic experiment were performed. In the first the dose of irradiation was 500 r instead of 400 r. In the second treatment was begun either on the day of, or 10 days after, transplantation of the tumour.

### *Propagation of the tumours*

Six different tumours were used. All were spontaneous mammary carcinomas which developed spontaneously in A-strain female mice and were maintained by transplantation every 2 to 3 weeks in females of the same strain.

One was used up to its seventh transplant generation but showed no evidence of deletion of tumour-specific antigens (see Woodruff and Symes, 1962*b*) ; the others were all in either their first or second transplant generation.

* Preliminary tests showed that the behaviour of the tumour in male and female recipients was indistinguishable.

*Irradiation*

The mice were irradiated in perspex boxes with a 230 kv Westinghouse machine (15 ma., 0·5 mm. Cu + 1 mm. Al, half-value layer 1·2 mm. Cu, focus-skin distance 75 cm.) under conditions of maximum back scatter. The dose rate was 66 r/min., measured in air at the surface of the animal nearest the tube.

*Immunization of cell donors*

Adult CBA mice were immunized by being given at one operation a subcutaneous transplant of the A-strain tumour to each flank and a third transplant to the peritoneal cavity in the form of an injection of tumour cell suspension. The spleen, and the axillary and inguinal lymph nodes on each side, were removed five days later and cell suspensions were prepared from them. Preliminary experiments showed that following this procedure cells from the spleen were about as effective as cells from the nodes both as regards their anti-tumour activity and their capacity to produce graft-versus-host disease in irradiated A-strain mice, whereas after immunization with subcutaneous transplants only spleen cells were much less effective than cells from the nodes.

*Preparation and injection of cell suspensions*

Cell suspensions were prepared by cutting up the spleen or the nodes with scissors, grinding the tissue very gently in Hanks's solution, using a hand-operated glass homogenizer, and straining through stainless steel mesh. The cells were spun down (200 **g** for 5 minutes) and resuspended in sufficient Hanks's solution to give a total nucleated cell count of 200 million cells/ml.

Doses of 100 million to 150 million cells were given in one injection ; larger doses were subdivided, 100–150 million cells being given morning and afternoon for either one or two days. All injections were given slowly into a tail vein.

*Administration of A-methopterin*

A-methopterin (Methotrexate) was given in the form of a freshly prepared aqueous solution by subcutaneous injection on the side of the body opposite to the tumour. The dose was 1·5 $\mu$g. per g. body weight every 2 days for 6 doses. The first dose was given 2, 4 or 6 days after the first cell injection.

### RESULTS

The results of the basic experiment are summarized in Table I.

It will be seen that irradiation alone produced a temporary arrest in the growth of the tumour, but did not alter the mean survival of the animal or the size of the tumour at death (Fig. 1 and 2) as compared with the values obtained in untreated controls.

Injection of 100 million isogeneic cells in addition to irradiation had no appreciable effect.

Injection of 100–150 million normal allogeneic cells increased the time for which tumour growth was arrested, and the tumour remained on average slightly smaller than in mice which received either no treatment or irradiation alone, but the mean survival of the animals remained the same. Injection of 600 million

TABLE I.—*The Effect of Irradiation and Intravenous Injection of Immunologically Competent Cells on a Transplanted Mammary Carcinoma and its Host. Treatment was begun Five Days After Transplantation of the Tumour*

| Treatment: Category | Treatment: Total cell dosage (millions) | Treatment: Interval between start of treatment and first dose of A-methopterin (days) | No. of animals | Period during which tumour showed no increase in size following treatment (days): Individual values | Period (days): Mean | Survival of animal (days): Individual values | Survival (days): Mean | Diameter of tumour at death (mm.): Individual values | Diameter (mm.): Mean |
|---|---|---|---|---|---|---|---|---|---|
| Controls . . (No treatment) | — | — | 25 | — | — | 68, 69, 48, 21, 21, 59, 38, 33, 49, 36, 41, 46, 34, 25, 26, 42, 51, 45, 55, 32, 49, 27, 42, 57, 27 | 41·6 | 25, 28, 27, 27, 20, 26, 29, 20, 31, 20, 29, 29, 26, 22, 24, 24, 19, 32, 30, 20, 32, 29, 24, 29, 25 | 25·8 |
| Irradiation only . | — | — | 26 | 0, 13, 13, 8, 9, 9, 9, 10, 10, 10, 4, 4, 9, 9, 0, 7, 9, 0, 11, 15, 5, 10, 9, 11*, 9, 8 | >8·1 | 42, 33, 40, 30, 42, 46, 31, 58, 55, 42, 46, 59, 24, 54, 37, 40, 53, 50, 49, 55, 49, 32, 55, 16, 45, 45 | 43·4 | 26, 19, 28, 18, 26, 25, 27, 32, 27, 29, 28, 30, 17, 22, 24, 23, 23, 22, 25, 26, 27, 17, 20, 3, 25, 30 | 23·8 |
| Irradiation + Isogeneic cells | 100 | — | 6 | 10, 10, 10, 10, 0, 0 | 6·7 | 31, 55, 41, 32, 33, 38 | 38·3 | 23, 26, 30, 23, 25, 27 | 25·7 |
| Irradiation + Normal allogeneic cells | 100–150 | — | 12 | 9, 17, 8, 15, 11, 17, 13, 12, 12, 12, 12, 14 | 12·7 | 42, 47, 50, 49, 56, 35, 27, 38, 58, 51, 35, 36 | 43·7 | 20, 21, 25, 24, 23, 18, 14, 27, 31, 28, 21, 11 | 21·9 |
| | 600 | — | 9 | 14, 14, 16, 10, 28, 10*, 21, 14, 21 | >16·5 | 61, 56, 36†, 20, 58, 15, 59, 55, 59 | 47·8 | 25, 25, 25, 14, 24, 1, 25, 28, 26 | 21·0 |
| Irradiation + Normal allogeneic cells + A-methopterin | 600 | 4 | 9 | 25*, 5, 19*, 12, 21*, 8, 13*, 14*, 14* | >14·6 | 30, 45, 24, 61, 26, 28, 18, 19, 19 | 30·0 | 4, 25, 3, 30, 3, 11, 3, 3, 2 | 9·3 |
| Irradiation + Immune allogeneic cells | 100 | — | 8 | 9*, 10*, 17, 15*, 11*, 12, 8*, 13*, | >11·9 | 14, 15, 64, 20, 16, 40, 13, 18 | 25·0 | 2, 0, 27, 1, 2, 27, 3, 2 | 8·0 |
| | 200–250 | — | 7 | 9*, 17*, 16, 15*, 8, 8, 13 | >12·3 | 14, 22, 53, 20, 45, 30, 45 | 32·7 | 2, 4, 31, 3, 25, 27, 27 | 17·0 |
| Irradiation + Immune allogeneic cells + A-methopterin | 200–250 | 2 | 5 | 13*, 16, 5, 22, 5 | >12·2 | 18, 58, 40, 55, 58 | 45·8 | 3, 24, 26, 30, 30 | 22·6 |
| | 200–250 | 4 | 19 | 13*, 32*, 9, 10, 17, 9, 36, 15, 6, 7, 12, 15, 25*, 13*, 32, 19*, 14*, 27*, 17* | >17·8 | 18, 37, 46, 56, 62, 14, 51, 50, 25, 12, 34, 67, 30, 18, 36, 25, 19, 32, 23 | 34·5 | 2, 3, 27, 30, 27, 4, 8, 22, 12, 2, 12, 25, 4, 3, 4, 3, 3, 3, 2 | 10·3 |
| | 200–250 | 6 | 8 | 7*, 11*, 9*, 16, 11*, 10, 10, 14* | >11·0 | 12, 16, 14, 58, 16, 61, 77, 19 | 34·1 | 0, 2, 2, 24, 0, 28, 30, 0 | 10·8 |

Dose of irradiation = 400 r.

Dose of A-methopterin = 1·5 μg./g. every 2 days for 6 doses.

* Animal died while tumours still showed no increase in size following treatment. Means based on figures which include such entries are prefixed by the sign >.

† Animal killed. Figures omitted in calculating mean.

normal allogeneic cells resulted in still more prolonged arrest of tumour growth and a slight increase in the mean survival time, but, with the exception of one animal which survived for only 10 days, all the mice died with large, actively growing tumours. Administration of A-methopterin, starting 4 days after the first spleen cell injection, did not weaken the anti-tumour effect, but far from increasing the mean survival time of the animals actually shortened it.

Tumours whose growth remained arrested following injection of normal allogeneic cells until the animal died all showed extensive necrosis, often with calcification, and varying degrees of fibrosis. All contained viable tumour cells, varying in amount from scattered single cells and minute clumps to small islets up to about 1 mm. diameter. Some sections showed massive plasma cell infiltration.

Irradiation and injection of allogeneic cells from immunized donors without other treatment had a marked inhibitory effect on the tumour and in 9 out of 15 animals growth remained completely arrested up to the time of death (Fig. 1 and 3), but the mean survival time was less than in the untreated controls. Administration of A-methopterin starting 2, 4 or 6 days after the irradiation and cell injection, on average slightly increased the mean survival time without weakening the anti-tumour effect. The figures suggest that the earlier this administration was started the greater the increase in survival and the less the damage to the tumour, but the evidence is not sufficient to warrant a firm conclusion to this effect.

Tumours whose growth remained arrested following injection of immune allogeneic cells up to the time of death again showed extensive necrosis (Fig. 3) and varying degrees of fibrosis and plasma cell infiltration (Fig. 4 and 5). In 3 animals no viable tumour cells could be found ; the remainder showed scattered minute clumps or small islets.

Increasing the dose of irradiation from 400 r to 500 r in mice which received no other treatment made no significant difference to the effect on the tumour or the survival of the animal. When irradiation was combined with the injection of 100 million normal allogeneic spleen cells, increasing the irradiation dose from 400 r to 500 r resulted in a slightly greater effect on the tumour, as judged by the time for which growth was arrested, but the mean survival of the animals was not significantly altered.

With either irradiation (400 r) alone, or irradiation (400 r) combined with injection of 100 million normal allogeneic spleen cells, treatment started on either

### EXPLANATION OF PLATES

FIG. 1.—These two mice each received a tumour transplant 20 days previously. The one on the left, which was treated by irradiation (400 r) alone, has a large tumour ; the one on the right, which was treated by irradiation and transplantation of immune allogeneic cells, does not have a palpable tumour but shows evidence of graft-versus-host reaction.

FIG. 2.—Active tumour from a mouse treated 49 days previously by irradiation only. H. and E. ×75.

FIG. 3.—Necrotic tumour from a mouse treated 16 days previously by irradiation and transplantation of immune allogeneic spleen cells. H. and E. ×155.

FIG. 4.—Tumour from a mouse treated 15 days previously by irradiation and transplantation of immune allogeneic cells, showing focal accumulation of plasma cells. H. and E. ×127.

FIG. 5.—Higher power view of the tumour shown in Fig. 4. H. and E. ×360.

FIG. 6.—Section of spleen from the same mouse as Fig. 2. Pyronin methyl-green. ×127.

FIG. 7.—Section of spleen from the same mouse as Fig. 3, showing extensive necrosis due to graft-versus-host reaction. Pyronin methyl-green. ×127.

1

2

3

Woodruff and Symes.

4

5

6

7

Woodruff and Symes.

the day the tumour was transplanted or 10 days after this had much the same effect as regards retardation of growth of the tumour as treatment started 5 days after tumour transplantation. There was no significant difference in the mean survival time of animals treated on day 5 or day 10 and the untreated controls, but irradiation on the day of tumour transplantation reduced the mean period of survival to 28 days, which is significantly less than that of the controls ($t = 2{\cdot}90$, $n = 29$, $P < 0{\cdot}01$).

### CONCLUSIONS AND DISCUSSION

It has been established that the growth of a transplanted mammary carcinoma may be greatly retarded, and the tumour may sometimes be completely destroyed, by exposing the recipient to a sublethal dose of whole body irradiation and then injecting allogeneic lymphoid cells. It is clear moreover that the cells played an essential role since irradiation alone, in the dosage used, was much less effective, whereas increasing the cell dose or using cells from immunized donors in place of normal cells increased the period of tumour growth arrest.

It has been reported previously by Defendi and Koprowski (1959) that transplantation of allogeneic lymphoid tissue to newborn hamsters reduced the incidence of tumours following a subsequent injection of polyoma virus, and more recently Wigzell (1961) has shown that growth of lymphoma cells transplanted to F1 hybrid mice from one parent strain could be inhibited by injection of normal lymphoid cells from the other parent strain 5 days previously, or of lymphoid cells from pre-immunized members of this strain at the same time as the lymphoma cell injection. The results now reported differ in that striking inhibition of growth was obtained with tumours of a type which appear to be much less susceptible to immunological attack than the lymphomas, and which had been transplanted 5 or 10 days before treatment was started.

None of the procedures tested, however, significantly increased the mean survival of the tumour-bearing animals, and some actually shortened it, because animals which were not killed by their tumour died instead as a result of the treatment (Fig. 1).

Many of these deaths were undoubtedly caused by the graft-versus-host reaction (Fig. 7. See Fig. 6 for comparison).

The possibility that some of the early deaths were due instead to rejection of the grafted cells combined with failure of regeneration of the recipient's own haemopoietic tissue, and were thus a manifestation of the sublethal zone effect described by Trentin (1958), cannot be excluded on the basis of available histological findings since these reveal only the terminal picture which may be indistinguishable in the two conditions ; it seems unlikely however in view of the relatively low dose of irradiation and high dose of immunologically competent cells* used in the present experiments.

The therapeutic efficiency of the procedure would be increased if a method could be found of " rescuing " the tumour-bearing animal after the foreign cells had produced an adequate effect on the tumour, or alternatively if the attack could

* In comparing these experiments using spleen cells with those of Trentin and others in which bone marrow was used, allowance must be made for the fact that in the spleen a much higher proportion of the nucleated cells are immunologically competent, as judged by their capacity to cause a graft-versus-host reaction.

be " focused " in such a way as to produce relatively more damage to the tumour and less to the host.

As mentioned in the introduction, there is evidence (Woodruff, 1962) that administration of A-methopterin may reduce the mortality resulting from injection of large doses of CBA strain spleen cells to sublethally irradiated normal (i.e. non-tumour bearing) A-strain mice, it therefore seemed possible that the same procedure might be effective as a rescue procedure in the present experiments, at any rate after injection of cells from non-immunized donors, but this expectation has not been fulfilled. Another procedure, which is currently being investigated, is the transplantation of isogeneic splenic tissue at various times after the irradiation and injection of allogeneic cells.

The possibility of " focusing " the immunological attack on the tumour is also being studied. The most obvious procedure would seem to be to study the effect of local treatment in the forms of injection of lymphoid cells and small pieces of tissue into and around an accessible tumour, or intraperitoneally in the case of ascites tumours. In experiments with large animals the injection might be given into the main arterial supply of the tumour. A subtle approach to the problem, based on a notion originally put forward by Levi, Schechtman, Sherings and Stanley (1959) in relation to the development of anti-cancer sera, is to use cells from animals previously made tolerant of normal tissue from the prospective recipient and later immunized against the tumour.

We are planning also to study the extent to which the accumulation of systemically injected immunologically competent cells in a tumour is modified by previous local treatment in the form of irradiation or injection of cytotoxic drugs ; and, conversely, to seek for methods by which accumulation of such cells in the spleen may be prevented or reduced.

The results of these investigations will be reported later. Meanwhile it has seemed justifiable to use normal allogeneic human spleen cells, obtained from spleens removed on account of idiopathic thrombocytopenic purpura and other diseases, for the treatment of patients with advanced cancer (Woodruff and Nolan, unpublished), and the possibility of obtaining immune cells by mutually immunizing two such patients against each other's tumour is under consideration. The first essential in such trials must be to avoid harm to the patient. It is necessary, therefore, to start with a small dose of irradiation (or radiomimetic drug) and a small dose of cells, and to increase these only in the light of accumulated clinical experience and all the available experimental data.

### SUMMARY

Experiments are described which show that the growth of a transplanted mammary carcinoma in A-strain mice may be greatly retarded, and the tumour may sometimes be completely destroyed, by exposing the recipient to a sublethal dose of irradiation (capable itself of producing only a very slight effect on the tumour) and then injecting allogeneic lymphoid cells from either a normal CBA mouse or a CBA mouse immunized against the A-strain tumour. These procedures did not increase (and sometimes decreased) the mean survival of the tumour-bearing animals because those which were not killed by their tumour died as a result of the graft-versus-host reaction. Attempts to prevent this fatal complication by administration of A-methopterin were unsuccessful, but other possible approaches to the problem are suggested.

This work was supported by a generous grant from the British Empire Cancer Campaign, of which grateful acknowledgement is made. One of us (M. O. S.) is a Medical Research Council Scholar and is indebted to the Council for this support.

We are grateful to Mr. George Brooks, Mrs. Y. H. S. Slater and Mr. Norman Samuel for expert technical help.

### REFERENCES

Barnes, D. W. H., Corp, M. Y., Ilbery, P. L. T., Loutit, J. F. and Neal, F. E.—(1959) ' Proc. 3rd Canadian Cancer Res. Conference '. New York (Academic Press, Inc.), p. 367.

*Idem*, Corp, M. Y., Loutit, J. F. and Neal, F. E.—(1956) *Brit. med. J.*, ii, 626.

*Idem* and Loutit, J. F.—(1957) *Brit. J. Haemat.*, **3**, 241.

Barrett, M. K.—(1955) In ' Origins of Resistance to Toxic Agents '. Edited by M. G. Sevag, R. D. Reid and O. E. Reynolds. New York (Academic Press, Inc.), p. 308.

Billingham, R. E. and Brent, L.—(1957) *Proc. roy. Soc.* B, **146**, 78.

*Idem*, Brent, L. and Medawar, P. B.—(1956) *Phil. Trans.* B, **239**, 357.

*Idem* and Silvers, W. K.—(1961) *Transplantation Bull.*, **28**, 493/113.

Black, M. M. and Speer, F. D.—(1959) *Int. Abstr. Surg.*, **109**, 105.

Defendi, V. and Koprowski, H.—(1959) *Nature, Lond.*, **184**, 1579.

Ewing, J.—(1941) ' Neoplastic Diseases '. 4th ed. Philadelphia (W. B. Saunders Co.), p. 587.

Finney, J. W., Byers, E. H. and Wilson, R. H.—(1960) *Cancer Res.*, **20**, 351.

Foley, E. J.—(1958) *Ibid.*, **13**, 835.

Koldovský, P.—(1961) *Folia biol.*, **7**, 157.—(1962) *Ibid.*, **8**, 90.

*Idem* and Lengerová, A.—(1960) *Ibid.*, **6**, 441.

Koller, P. C. and Doak, S. M. A.—(1959) *Int. J. Radiol. biol.*, Spec. Suppl. 327.

Levi, E., Schechtman, A. M., Sherins, R. S. and Stanley, T.—(1959) *Nature, Lond.*, **184**, 563.

Lewis, M. R. and Aptekman, M. B.—(1952) *Cancer Res.*, **5**, 411.

Mathé, G.—(1960) *Blood*, **16**, 1073.

*Idem* and Bernard, J.—(1959*a*) *Sang*, **30**, 789.—(1959*b*) *Rev. franç. Etudes clin. biol.*, **4**, 442.

*Idem*, Bernard, J., Schwarzenberg, L., Larrieu, M. J., Lalanne, C., Dutreix, A., Denoix, P., Surmont, J., Schwarzmann, V. and Céoara, B.—(1959) *Ibid.*, **4**, 675.

*Idem*, Bernard, J., De Vries, M. J., Schwarzenberg, L., Larrieu, M. J., Lalanne, C. M., Dutreix, A., Amiel, J. L. and Surmont, J.—(1960) *Rev. Hémat.*, **15**, 115.

Mitchison, N. A.—(1955) *J. exp. Med.*, **102**, 157.

Prehn, R. T.—(1960) *Cancer Res.*, **20**, 1614.

*Idem* and Main, J. M.—(1957) *J. nat. Cancer Inst.*, **18**, 769.

Révész, L.—(1960) *Cancer Res.*, **20**, 443.

Russell, P. S.—(1962) ' Modification of Runt Disease in Mice by Various Means '. In Ciba Symposium on Transplantation. London (J. & A. Churchill), p. 350.

Stern, K.—(1960) *Nature, Lond.*, **185**, 787.

Trentin, J. J.—(1958) *Ann. N.Y. Acad. Sci.*, **73**, 799.

Wigzell, H.—(1961) *Cancer Res.*, **21**, 365.

Woodruff, M. F. A.—(1960) ' The Transplantation of Tissues and Organs '. Springfield, Ill. (C. C. Thomas).—(1962) *Nature, Lond.*, **195**, 727.

*Idem* and Symes, M. O.—(1962*a*) *Brit. J. Cancer*, **16**, 120.—(1962*b*) *Ibid.*, **16**, 484.